# *Roegneria yenchiana*: A new species in the Triticeae (Poaceae) from the Hengduan Mountain region

**DOI:** 10.1002/ece3.11171

**Published:** 2024-03-17

**Authors:** Li‐Na Sha, Xiao Liang, Xin‐Yi Zhang, Shan Gao, Yue Zhang, Yong‐Hong Zhou, Xing Fan

**Affiliations:** ^1^ College of Grassland Science and Technology Sichuan Agricultural University Chengdu Sichuan China; ^2^ Triticeae Research Institute Sichuan Agricultural University Chengdu Sichuan China

**Keywords:** *Elymus*, genome, phylogenetic analysis, *Roegneria*, **StY** genomes

## Abstract

*Roegneria yenchiana* sp. nov. (Triticeae) is a new species collected from Shangri‐la of Yunnan Province in China based on morphological, cytological, and molecular data. It is morphologically characterized by one spikelet per node, rectangular glums, awns flanked by two short mucros in lemmas, distinguished from other species of *Roegneria*. The genomic in situ hybridization results indicate that *R. yenchiana* is an allotetraploid, and its genomic constitution is **StY**. Phylogenetic analyses based on multiple loci suggested that *R. yenchiana* is closely related to *Pseudoroegneria* and *Roegneria,* and the *Pseudoroegneria* served as the maternal donors during its polyploid speciation.

## Graphical abstract

Roegneria yenchiana sp. nov. (Triticeae) is described as a new species collected from the Hengduan Mountain Region. Roegneria yenchiana contains the StY genomic constitution. Pseudoroegneria served as the maternal donors during the polyploid speciation of Roegneria yenchiana.

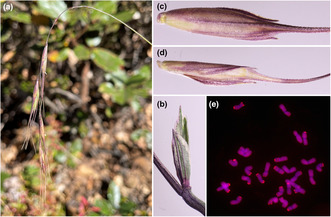

## 1 INTRODUCTION

*Roegneria* C, Koch was erected and circumscribed by Koch (1848) according to *Roegneria caucasica* C. Koch as the type species. Nevskis (1934) played a major role in popularizing the genus *Roegneria*, which he recognized distinct from *Agropyron* Gaertner as an independent genus, and described 49 species of *Roegneria*. Chen and Zhu (2006) and Tvelev (1976) included *Roegneria* in *Elymus* because of their many commonly morphological characters, e.g., plants usually tufted; lemma lanceolate‐oblong, rounded abaxially, 5‐veined, veins connivent at apex. Despite the criteria used, there is morphological disagreement over the circumscription of *Elymus* and *Roegneria*, and *Roegneria* is distinguished from *Elymus* by single spikelet per rachis node with lanceolate glumes and short and broader palea as well as glume retention after caryopsis abscission (Baum et al., 1991, 1995; Yen & Yang, 2011). Based on these morphological disagreements, Baum et al. (1991) specify only one genus, *Roegneria*. Cytogenetics and molecular phylogeny provided additional support for *Roegneria* as an independent genus (Fan et al., 2013; Lu et al., 1988; Yen & Yang, 2011), corresponding to the genome system of classification in Triticeae (Dewey, 1984). Since then, *Roegneria* is a large genus in the wheat tribe (Poaceae: Triticeae), which includes approximately 100 species that are of Asian origin and distributed in the Qinghai–Tibetan Plateau, Central Asia, East Asia, West Asia and South‐east Europe (Yen & Yang, 2011). The largest number is found mainly in the mountains of the Qinghai–Tibetan Plateau, with 33 species being recorded. Most species of *Roegneria* can usually be recognized in the field by a combination of characters: (a) lack of underground rhizomes; (b) spikes often slightly curved to erect, with arrangement of spikelets nearly parallel to and appressed to rachis and only one per node, with long rachis internodes; (c) spikelets functionally disarticulating above the glumes (Baum et al., 1995). Cytologically, all the species of *Roegneria* are allotetraploids with **StY** genomes (Yen & Yang, 2011). The **St** genome is derived from *Pseudoroegneria* (Nevski) Á. Löve (Löve, 1984; Wang et al., 1994). It is unknown where the **Y** genome originates, although it is a fundamental *Roegneria* genome (Yen & Yang, 2011). The tetraploid species *Roegneria alashanica* Keng was found to have the **StSt** ^**Y**^ genome constitution (Wang & Jensen, 2017), suggesting that the **Y** genome might derive from **St** through **StY**. Dewey (1984) considered that the **Y** genome has its origin in Central Asia or the Himalaya region, and may be extinct. Extensive cytogenetic and molecular studies have suggested that the **St** (*Pseudoroegneria*), **W** [*Australopyrum* (Tzvelev) Á. Löve], **V** [*Dasypyrum villosum* (L.) Candargy], and **Xp** [*Peridictyon* O. Seberg, S. Frederiksen & C. Baden] genomes are potential donors of the **Y** genome (Fan et al., 2013; Lei et al., 2022; Liu et al., 2006, 2022; Sun & Komatsuda, 2010).

Here, we described a new species of *Roegneria* discovered from the Hengduan Mountain Region during an expedition to the Qinghai‐Tibetan plateau in 2021 to collect germplasm of various members of the Triticeae. The new species was discovered in stony slope of Haba Snow Mountain at altitude about 3200 m and 2500 m, and in stony slope of Yulong Snow Mountain at an altitude of approximately 3200 m. The two localities are more than 90 km apart. Using multiple sampling of the new species, we recognized it with morphological observations, genomic in situ hybridization (GISH), and phylogenetic reconstruction methods. Our goal was to determine whether this was the case as a new species within *Roegneria* and, if so, to determine the genomic constitution of the new species and provide it with an appropriate name.

## 2 MATERIALS AND METHODS

### 2.1 Plant sampling

Three accessions of the new species were sampled, with each accession representing one sample point based on distinct locality and/or altitude (Table S1). Twelve **StY** genome species from the genus *Roegneria* and 33 diploid taxa representing 19 basic genomes in Triticeae were included in this study, and their DNA sequences for phylogenetic analyses were obtained from published data (Fan et al., 2013; Huang et al., 2002; Lei et al., 2022; Mason‐Gamer, 2004) (Table S1). The plants and voucher specimens are deposited at the Herbarium of Triticeae Research Institute, Sichuan Agricultural University, China (SAUTI).

### 2.2 Morphological observation

Morphological features including underground rhizomes, stalks, cauline internodes, leaf auricles and blades, glumes, lemmas, paleas, awns, and anthers were observed. At least 20 measurements on fresh material were performed on each morphological variable by using a stereomicroscope (Olympus SZX7, Tokyo, Japan) with a digital camera.

### 2.3 Genomic in situ hybridization (GISH)

Roots of the new species were collected from germinating seeds and adult plants, treated with nitrous oxide for 2.5 h, and fixed in 90% glacial acetic acid for 5 min. Chromosome preparations were performed using drop methods, according to Tan et al. (2021). The genomic DNA was isolated from fresh leaves by the CTAB method and then labeled with the DIG‐Nick Translation Kit (Roche, Indianapolis, IN, USA). *Roegneria ciliaris* with the **StY** genomic constitution was used as a probe. The hybridization procedure, detection, and visualization methods follow those described by Tan et al. (2021). Images were captured with an Olympus BX51 fluorescence microscope (Japan).

### 2.4 Phylogenetic analysis

Phylogenetic analysis is routinely applied to illustrate evolutionary and taxonomic questions. We carried out phylogenetic analysis for the new species and its affinitive species within the Triticeae based on three unlinked single‐copy nuclear genes (*Acc1*, plastid Acetyl‐CoA carboxylase; *DMC1*, disrupted meiotic cDNA; *GBSSI*, Granule‐Bound Starch Synthase I) and three chloroplast regions [*trn*L‐F, *trn*L (UAA)‐*trn*F (GAA); *mat*K, maturase coding gene; *rbc*L, ribulose‐1, 5‐bisphosphate carboxylase/oxygenase]. Prior to phylogenetic analysis, The *Acc1*, *DMC1*, *GBSSI*, *trn*L‐F, *mat*K, and *rbc*L sequences were amplified by polymerase chain reaction (PCR) using the primers listed in Table S2 under cycling conditions reported previously (Sha et al., 2016, 2017), and PCR products were cloned into the pMD18‐T vector (TaKaRa, Dalian, China) following the manufacture's instruction. At least 10 random independent clones were selected for commercial sequencing. For each gene fragment, in cases when multiple identical sequences resulted from cloned PCR products of each accession, only one sequence was included in the data matrix.

Multiple sequence alignment was conducted using ClustalX (Thompson et al., 1999), with default parameters and additional manual edits to minimize gaps. Phylogenetic analyses were conducted using maximum likelihood (ML) and Bayesian inference (BI). ML analysis was performed using RAxML v8.2.8 under the GTR + GAMMA model on the XSEDE supercomputer at the CIPRES Science Gateway platform (Miller et al., 2010). Analyses included inference of the ‘best tree’ and generation of 1000 bootstrap replicates to obtain node support measures. BI analysis was conducted with MrBayes v3.2.7a under the same evolutionary model and supercomputer platform (Miller et al., 2010) as ML analysis. Four MCMC (Markov Chain Monte Carlo) chains were run for 2,000,000 generations. Trees were sampled every 1000 generations until reaching the convergence parameters (standard deviation less than 0.01). The first 25% of generated trees representing the burn‐in phase were discarded, and the remaining trees were used to construct the 50%‐majority rule consensus trees. The statistical confidence in nodes was evaluated by posterior probabilities (PP). PP‐value less than 90% was not included in the figures.

## 3 RESULTS

### 3.1 Morphological characters

**FIGURE 1:**
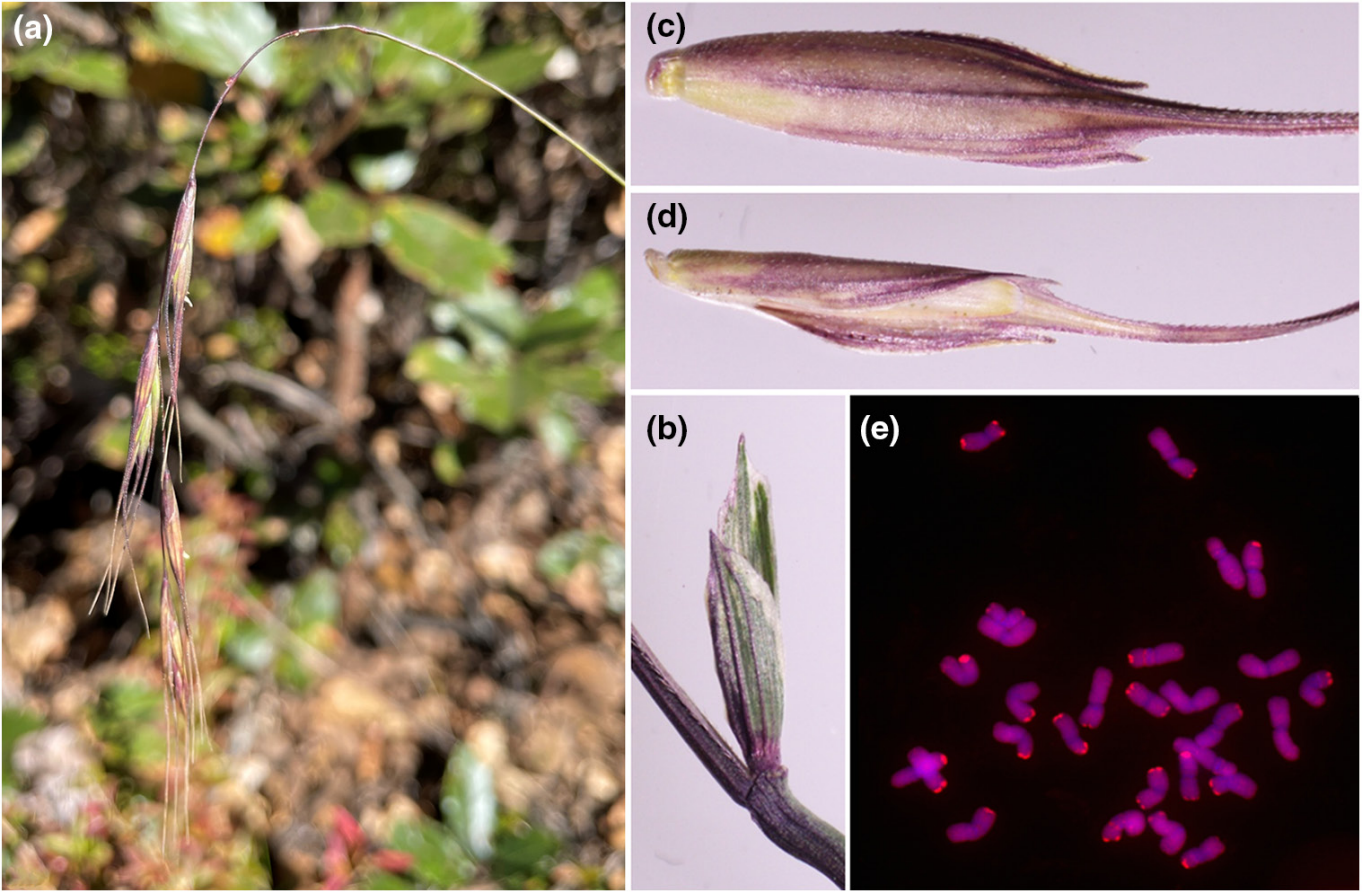
Features of *Roegneria yenchiana*. (a) Inflorescences; (b) Glumes; (c) Lemmas; (d) Palea; (e) Genomic in situ hybridization photographs of mitotic metaphase in *Roegneria yenchiana* display red fluorescence when probed with *R. ciliaris*, indicating *R. yenchiana* has the **StY** genomes.

*Roegneria yenchiana* is morphologically recognized as a species of *Roegneria* by the typical characters that have traditionally one spikelet per node and spikelets with functionally disarticulating above the glumes. This species is distinguished from other species of *Roegneria* by its rudimentary spikelets at the tortuous inflorescence base, rectangular glumes, and awns flanked by two short mucros in lemmas (Figure 1a–d). *Roegneria ciliaris* occurring in the Hengduan Mountain region has the **StY** genomes (Lu, 1992), which is morphologically the most similar species to *R. yenchiana*. Despite this, *R. ciliaris* has not been observed near to the place where *R. yenchiana* grows. *Roegneria yenchiana* can be distinguished from *R. ciliaris* in several traits, including glume length, ciliates in lemma margin, lemma back, and the paleas length.

### 3.2 GISH

GISH was carried out with the **StY** genome species (from *Roegneria ciliaris*) being used as a probe. GISH results showed that *R. yenchiana* has 28 chromosomes, and all the chromosomes displayed **StY** genome hybridization signal along their entire chromosomal length (Figure 1e). Thus, the genomic constitution of *R. yenchiana* is **StStYY**.

### 3.3 Phylogenetic analyses

Three unlinked low‐copy nuclear gene (*Acc*1, *DMC*1, and *GBSSI*) and three chloroplast region (*trn*L‐F, *mat*K, and *rbc*L) sequences were separately amplified and sequenced for all the accessions of *R. yenchiana*. They were then analyzed with published **St**‐ and **Y**‐type sequences from twelve species of *Roegneria* and those from 33 diploid taxa representing 19 basic genomes in Triticeae. Consequently, six homoeologous sequences representing two distinct types (**St**‐ and **Y**‐type) of each nuclear gene and three sequences of each chloroplast region were detected from all the accession of *R. yenchiana*. Four datasets, including *Acc1* data, *DMC1* data, *GBSSI* data, and combined chloroplast (*trn*L‐F + *mat*K + *rbc*L) data, were used to conduct separately alignments and phylogenetic analyses.

**FIGURE 2:**
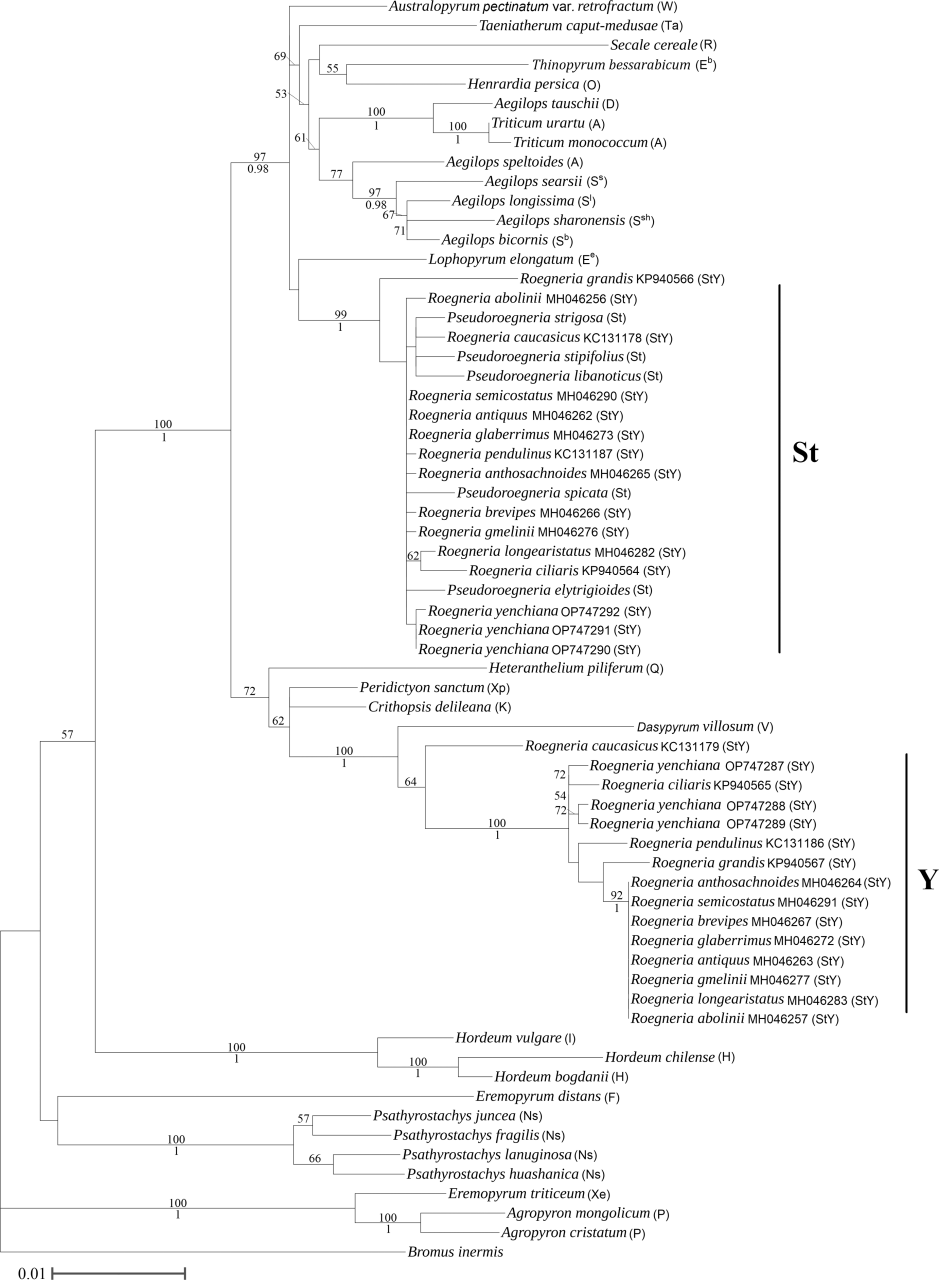
Phylogenetic tree inferred from the *Acc1* sequences of *Roegneria yenchiana* and its affinitive species within the Triticeae. Numbers above nodes are bootstrap support values ≥50%, and numbers below nodes are Bayesian posterior probability values ≥90%. Polyploid species names are followed by GenBank numbers. The upper case letters in parentheses indicate the genome type of the species.

The features of *Acc1* data, *DMC1* data, *GBSSI* data, and combined chloroplast data were summarized in Table S3. The aligned *Acc1* sequences yielded 1400 characters, of which 411 were variable characters and 201 were informative. ML analysis of the *Acc1* data yielded a single phylogenetic tree (−Lnlikelihood = 5890.5424). ML and Bayesian analyses of the *Acc1* data recovered the same topology. The tree illustrated in Figure 2 was the ML tree of bootstrap support (BS) above and posterior probabilities (PP) below branches. The *Acc1* sequences of *R. yenchiana* were represented in two clades, corresponding to the two genomic types (**St** and **Y**). The **St**‐type *Acc1* sequences of *R. yenchiana* were in one monophyletic group and then formed a paraphyletic grade with those **St**‐type sequences from other *Roegneria* and *Pseudoroegneria*. The **Y**‐type *Acc1* sequences of *R. yenchiana* and the sequence of *Roegneria ciliaris* formed a paraphyletic grade. Of the 959 total characters in the DMC1 data, 343 characters were variable, and 158 characters were informative. ML and Bayesian analyses of the *DMC1* data recovered the same topology. The ML tree (−Lnlikelihood = 4907.2580) inferred from the *DMC1* data showed that the **St**‐type and **Y**‐type sequences from the new species were split into two well‐supported clades (Figure S1). The **St**‐type *DMC1* sequences of *R. yenchiana* were scattered in different groups and clustered with those from different species of *Roegneria*. The **Y**‐type *DMC1* sequences of *R. yenchiana* formed one monophyletic group and grouped with the sequence of *Roegneria brevipes*. In the *GBSSI* sequence data matrix, of the 1104 total characters, 472 were variable, and 277 were parsimony informative. ML and Bayesian analyses of the *GBSSI* data recovered the same topology. In ML tree (−Lnlikelihood = 8424.8144) inferred from the *GBSSI* data, the **St**‐type *GBSSI* sequences of *R. yenchiana* formed one monophyletic group, and then clustered with one group including *Roegneria gmelinii* and *Roegneria pendulinus* (Figure S2). The **Y**‐type *GBSSI* sequences of *R. yenchiana* were in one monophyletic group and formed a paraphyletic grade with those **Y**‐type sequences from several species of *Roegneria* (*R. semicostatus*, *R. anthosachnoides*, and *R. ciliaris*). The combined *trn*L‐F, *mat*K, and *rbc*L data yielded 3089 characters of which 306 were variable characters and 132 were informative. ML and Bayesian analyses of the combined chloroplast data recovered the same topology. In the ML tree (−Lnlikelihood = 7088.8370) inferred from the combined chloroplast data, all the accessions of *R. yenchiana* formed one monophyletic group, and this group was clustered with the species from *Roegneria* and *Pseudoroegneria* (Figure S3).

### 3.4 Taxonomic treatment

**FIGURE 3:**
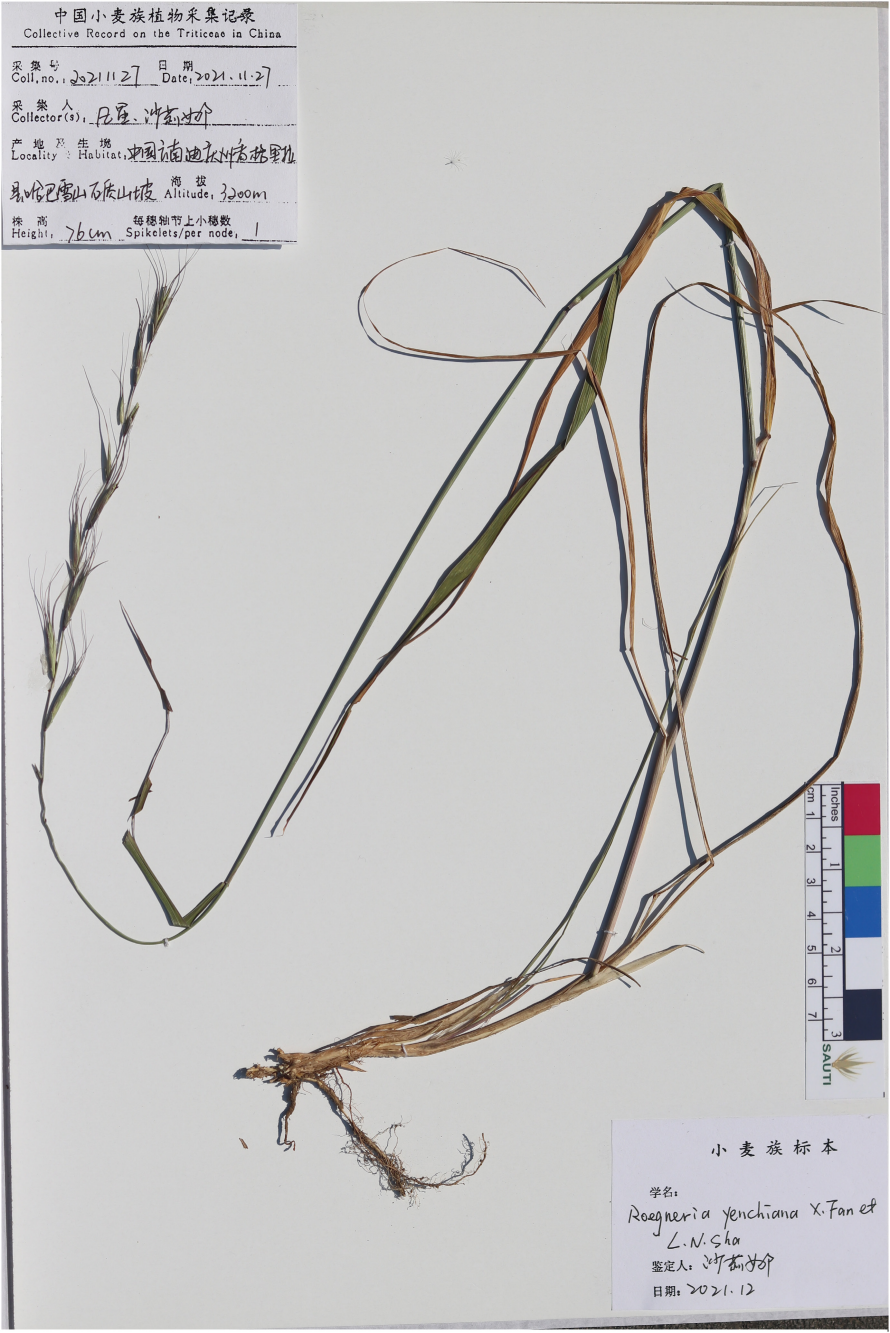
The holotype of the new species *Roegneria yenchiana* X. Fan et L. N. Sha.

***Roegneria yenchiana* X. Fan et L. N. Sha, sp. *nov***. (Figures 1 and 3)

TYPE: CHINA, **Yunnan**, Shangri‐la, Tiger Leaping Gorge, in the stony slope of Haba Snow Mountain, 3200 m, 11 November 2021, X. Fan & L.N. Sha 20,211,127 (holotype: SAUTI; isotype: SAUTI).

#### 3.4.1 Diagnosis

*Roegneria yenchiana* is morphologically the most similar species to *R. ciliaris* (**StY**) but is distinguished from *R. ciliaris* by its lower glume length (3–5 mm vs. 7–8 mm), ciliates in lemma margin (absent vs. about 1 mm), lemma back (scabrous or pubescent on lower parts vs. hispid), and the paleas length (slightly shorter than lemma vs. 2/3 the length of lemma).

#### 3.4.2 Description

Perennial herb, cespitose; culms usually erect, 50–100 cm tall, purplish at maturity. Leaf sheaths glabrous; ligule about 0.3–0.6 mm long, truncate or lacerate; blades flat, 27–40 cm long, 4–6 mm wide, glabrous. Spikes linear, inclined to nodding, purplish at maturity, 10–26 cm long (excluding awns), 5–10 mm wide, 7–16 spikelets per spike; 1 spikelets per node; rachises scabrous and margin shortly ciliolate; internodes 14–25 mm long; usually 1–4 rudimentary spikelets at the tortuous inflorescence base. Spikelets 18–25 mm (excluding awns) long, with 6–10 florets, disarticulation above the glumes, beneath each floret. Glums unequal, rectangular, oblique, margin membranous, usually lower glume 2/3 the length of upper glume, lower glum 3–5 mm, upper glume 5–8 mm, 3–5 prominent veins, sometimes setulose on upper veins, apex acuminate and with 1 tooth. Lemmas oblong‐lanceolate to oblong, scabrous, pubescent on lower parts, 3 veins, prominent midvein, first lemma 9–12 mm, awned, awns 17–25 mm, reflexed, awns flanked by 2 short teeth with about 0.5 mm long. Paleas oblong, slightly shorter than lemma, ciliolate along keels in the distal 1/3, apex truncate. Chromosome number 2*n* = 4*x* = 28; genome constitution **StStYY**.

#### 3.4.3 Phenology

Flowering: July–August and fruiting October–November.

#### 3.4.4 Distribution, habitat, and name

*Roegneria yenchiana* is known only from a few localities in the northwest of Yunnan, China. It was found among bushes or on stony mountain slopes between 2500 and 3500 m, growing together with *Campeiostachys nutans* (Griseb.) J. L. Yang, B. R. Baum et C. Yen and *Campeiostachys dahurica* var. *cylindrica* (Franch.) J. L. Yang, B. R. Baum et C. Yen. These two species were previously included in the genus *Elymus*. According to the genome system of classification, they were transferred into the genus *Campeiostachys* because of their **StYH** genome constitutions.

*Roegneria yenchiana* is named for commemorating Prof. Yen Chi, a biosystematic scientist of the Triticeae from the Triticeae Research Institute of Sichuan Agricultural University (SAU), China. He was evaluated as an important person in the taxonomic research of the Triticeae by Chairman Prof. Roland von Bothmer in the eighth international Triticeae symposium.

## 4 DISCUSSION

The morphological characters of *R. yenchiana* differ from all species previously described in *Roegneria*, especially in its rudimentary spikelets at the inflorescence base, rectangular glumes, and two short mucros in lemmas. GISH results confirmed that *R. yenchiana* is a tetraploid (2*n* = 4*x* = 28) with the **StY** genome constitution. Based on the genomic system of classification as a guide to resolve taxonomic treatment in Triticeae (Barkworth & von Bothmer, 2009; Baum et al., 1995; Dewey, 1984; Wang et al., 1994; Yen & Yang, 2011), this species should be included in the genus *Roegneria*. Phylogenetic analysis based on all three unlinked single‐copy sequences showed that the **St**‐genome homoeologous types of *R. yenchiana* were grouped with the sequences of *Pseudoroegneria* and *Roegneria*, and the **Y**‐genome homoeologous types of *R. yenchiana* were clustered with the sequences of *Roegneria* with high statistic supports (BS > 70% and PP > 0.9). In the phylogenetic tree inferred from the combined chloroplast sequences, all three accessions of *R. yenchiana* were grouped with *Pseudoroegneria* and *Roegneria* (BS = 81% and PP = 1.0). These results indicated that *Pseudoroegneria* and *Roegneria* are closely related to *R. yenchiana*. Combined with GISH analysis, it can be concluded that the **St** genome of *R. yenchiana* is derived from the genus *Pseudoroegneria*, and the *Pseudoroegneria* served as the maternal donors during its polyploid speciation.

## AUTHOR CONTRIBUTIONS

**Li‐Na Sha:** Conceptualization (equal); writing – original draft (equal); writing – review and editing (equal). **Xiao Liang:** Investigation (lead); methodology (equal). **Xin‐Yi Zhang:** Investigation (equal); methodology (equal). **Shan Gao:** Investigation (equal); methodology (equal). **Yue Zhang:** Investigation (equal); methodology (equal). **Yong‐Hong Zhou:** Conceptualization (equal); writing – review and editing (equal). **Xing Fan:** Conceptualization (equal); funding acquisition (lead); writing – review and editing (equal).

## FUNDING INFORMATION

The Second Tibetan Plateau Scientific Expedition and Research Program (STEP), Grant/Award Number: 2019QZKK0303; National Natural Science Foundation of China, Grant/Award Number: 31870360 & 32171603; the Transformation Project of Scientific and Technological Achievements of Qinghai Province, Grant/Award Number: 2023‐NK‐101; the Key Research and Development Program of Science and Technology Project of Sichuan Province, Grant/Award Number: 2021YFYZ0020.

## CONFLICT OF INTEREST STATEMENT

The authors declare no conflicts of interest.

## Supporting information


Figure S1.

Figure S2.

Figure S3.

Table S1.

Table S2.

Table S3.

## Data Availability

The sequences used in this study have been deposited in The National Center for Biotechnology Information (NCBI) database. GenBank accession numbers are provided in the supplementary file Table S1.

## References

[ece311171-bib-0001] Barkworth, M. E. , & von Bothmer, R. (2009). Scientific names in the Triticeae. In C. Feuillet & G. J. Muehlbauer (Eds.), Genetics and genomics of the Triticeae (pp. 3–30). Springer.

[ece311171-bib-0002] Baum, B. R. , Yang, J. L. , & Yen, C. (1995). Taxonomic separation of *Kengyilia* (Poaceae: Triticeae) in relation to nearest related *Roegneria*, *Elymus*, and *Agropyron*, based on some morphological characters. Plant Systematics and Evolution, 194, 123–132.

[ece311171-bib-0003] Baum, B. R. , Yen, C. , & Yang, J. L. (1991). *Roegneria*: Its generic limits and justification for its recognition. Canadian Journal of Botany, 69, 282–294.

[ece311171-bib-0004] Chen, S. L. , & Zhu, G. H. (2006). *Elymus* Linn. In Z. Y. Wu , P. H. Raven , & D. Y. Hong (Eds.), Flora of China (Vol. 22, pp. 400–429). Science Press; Missouri Botanical Garden Press.

[ece311171-bib-0005] Dewey, D. R. (1984). The genomic system of classification as a guide to intergeneric hybridization with the perennial Triticeae. In J. P. Gustafson (Ed.), Gene manipulation in plant improvement (pp. 209–279). Columbia University Press.

[ece311171-bib-0006] Fan, X. , Sha, L. N. , Dong, Z. Z. , Zhang, H. Q. , Kang, H. , Wang, Y. , Wang, X. L. , Zhang, L. , Ding, C. B. , Yang, R. W. , Zheng, Y. L. , & Zhou, Y. H. (2013). Phylogenetic relationships and Y genome origin in *Elymus* L. sensu lato (Triticeae; Poaceae) based on single‐copy nuclear *Acc1* and *Pgk1* gene sequences. Molecular Phylogenetics and Evolution, 69, 919–928. 23816902 10.1016/j.ympev.2013.06.012

[ece311171-bib-0007] Huang, S. X. , Sirikhachornkit, A. , Su, S. J. , Fairs, J. , Gill, B. , Haselkorn, R. , & Gornicki, P. (2002). Genes encoding plastid acetyl‐CoA carboxylase and 3‐phosphoglycerate kinase of the *Triticum*/*Aegilops* complex and the evolutionary history of polyploidy wheat. Proceedings of the National Academy of Sciences of the United States of America, 99, 8133–8138. 12060759 10.1073/pnas.072223799 PMC123033

[ece311171-bib-0008] Koch, K. (1848). Beiträge zu einer Flora des Orientes. Linnaea, 21(4), 289–736.

[ece311171-bib-0009] Lei, Y. X. , Fan, X. , Sha, L. N. , Wang, Y. , Kang, H. Y. , Zhou, Y. H. , & Zhang, H. Q. (2022). Phylogenetic relationships and the maternal donor of *Roegneria* (Triticeae: Poaceae) based on three nuclear DNA sequences (ITS, *Acc1*, and *Pgk1*) and one chloroplast region (*trn*L‐F). Journal of Systematics and Evolution, 60, 305–318.

[ece311171-bib-0010] Liu, Q. L. , Ge, S. , Tang, H. B. , Zhang, X. L. , Zhu, G. F. , & Lu, B. R. (2006). Phylogenetic relationships in *Elymus* (Poaceae: Triticeae) based on the nuclear ribosomal internal transcribed spacer and chloroplast *trn*L‐F sequences. New Phytologist, 170, 411–420. 16608465 10.1111/j.1469-8137.2006.01665.x

[ece311171-bib-0011] Liu, Q. L. , Liu, L. , Ge, S. , Fu, L. P. , Bai, S. Q. , Lv, X. , Wang, Q. K. , Chen, W. , Wang, F. Y. , Wang, L. H. , Yan, X. B. , & Lu, B. R. (2022). Endo‐allopolyploidy of autopolyploids and recurrent hybridization‐A possible mechanism to explain the unresolved Y‐genome donor in polyploid *Elymus* species (Triticeae: Poaceae). Journal of Systematics and Evolution, 60(2), 344–360.

[ece311171-bib-0012] Löve, À. (1984). Conspectus of the Triticeae. Feddes Repertorium, 95, 425–521.

[ece311171-bib-0013] Lu, B. R. (1992). *Elymus yangii* (Poacea, Triticeae), a new species from Tibet. Willdenowia, 22, 129–132.

[ece311171-bib-0014] Lu, B. R. , Yen, C. , & Yang, J. L. (1988). The studies of morphological variations and karyotype analysis on the three *Roegneria* species. Plant Diversity, 10, 139–146.

[ece311171-bib-0015] Mason‐Gamer, R. J. (2004). Reticulate evolution, introgression, and intertribal gene capture in an allohexaploid grass. Systematic Biology, 53, 25–37. 14965898 10.1080/10635150490424402

[ece311171-bib-0016] Miller, M. A. , Pfeiffer, W. , & Schwartz, T. (2010). Creating the CIPRES science gateway for inference of large phylogenetic trees. In: *Proceedings of the gateway computing environments workshop (GCE)*. New Orleans, pp. 1–8.

[ece311171-bib-0017] Nevskis, A. (1934). *Roegneria* C. Koch. In V. L. Komarov , R. Y. Rozhevits , & B. K. Shishkin (Eds.), Flora of the USSR (Vol. 2, pp. 599–627). Israel Program for Scientific Translations.

[ece311171-bib-0018] Sha, L. N. , Fan, X. , Li, J. , Liao, J. Q. , Zeng, J. , Wang, Y. , Kang, H. Y. , Zhang, H. Q. , Zheng, Y. L. , & Zhou, Y. H. (2017). Contrasting evolutionary patterns of multiple loci uncover new aspects in the genome origin and evolutionary history of *Leymus* (Triticeae; Poaceae). Molecular Phylogenetics and Evolution, 114, 175–188. 28533082 10.1016/j.ympev.2017.05.015

[ece311171-bib-0019] Sha, L. N. , Fan, X. , Zhang, H. Q. , Kang, H. Y. , Wang, Y. , Wang, X. L. , Yu, X. F. , & Zhou, Y. H. (2016). Phylogeny and molecular evolution of the *DMC1* gene in the polyploid genus *Leymus* (Triticeae: Poaceae) and its diploidrelatives. Journal of Systematics and Evolution, 54, 250–263.

[ece311171-bib-0020] Sun, G. L. , & Komatsuda, T. (2010). Origin of the Y genome in *Elymus* and its relationship to other genomes in Triticeae based on evidence from elongation factor G (*EF‐G*) gene sequences. Molecular Phylogenetics and Evolution, 56, 727–733. 20363342 10.1016/j.ympev.2010.03.037

[ece311171-bib-0021] Tan, L. , Zhang, H. Q. , Chen, W. H. , Deng, M. Q. , Sha, L. N. , Fan, X. , Kang, H. Y. , Wang, Y. , Wu, D. D. , & Zhou, Y. H. (2021). Genome composition and taxonomic revision of *Elymus purpuraristatus* and *Roegneria calcicola* (Poaceae: Triticeae) based on cytogenetic and phylogenetic analyses. Botanical Journal of the Linnean Society, 196, 242–255.

[ece311171-bib-0022] Thompson, J. D. , Plewniak, F. , & Poch, O. (1999). A comprehensive comparison of multiple sequence alignment programs. Nucleic Acids Research, 27, 2682–2690. 10373585 10.1093/nar/27.13.2682 PMC148477

[ece311171-bib-0023] Tvelev, N. N. (1976). Grasses of the Soviet Union. Zlaki SSSR. Nauka.

[ece311171-bib-0024] Wang, R. R.‐C. , Bothmer, R. V. , Dvorak, J. , Fedak, G. , Linde‐Laursen, I. , & Muramatsu, M. (1994). Genome symbols in the Triticeae (Poaceae). In: R.R.‐C. Wang, K.B. Jensen, & C. Jaussi. (Eds.), *Proc 2nd Intern. Triticeae Symp*, Logan, Utah, USA, pp. 29–34.

[ece311171-bib-0025] Wang, R. R.‐C. , & Jensen, K. B. (2017). *Roegneria alashanica* Keng: A species with the StStSt^Y^St^Y^ genome constitution. Genome, 60, 546–551. 28314108 10.1139/gen-2016-0216

[ece311171-bib-0026] Yen, C. , & Yang, J. L. (2011). Triticeae biosystematics (Vol. 4). Chinese Agricultural Press.

