# Supplementary figures and images for: Roegneria yenchiana: A new species in the Triticeae (Poaceae) from the Hengduan Mountain region
Source: Ecol Evol. 2024 Mar 17;14(3):e11171. doi: 10.1002/ece3.11171 (PMC10944672; doi:10.1002/ece3.11171)

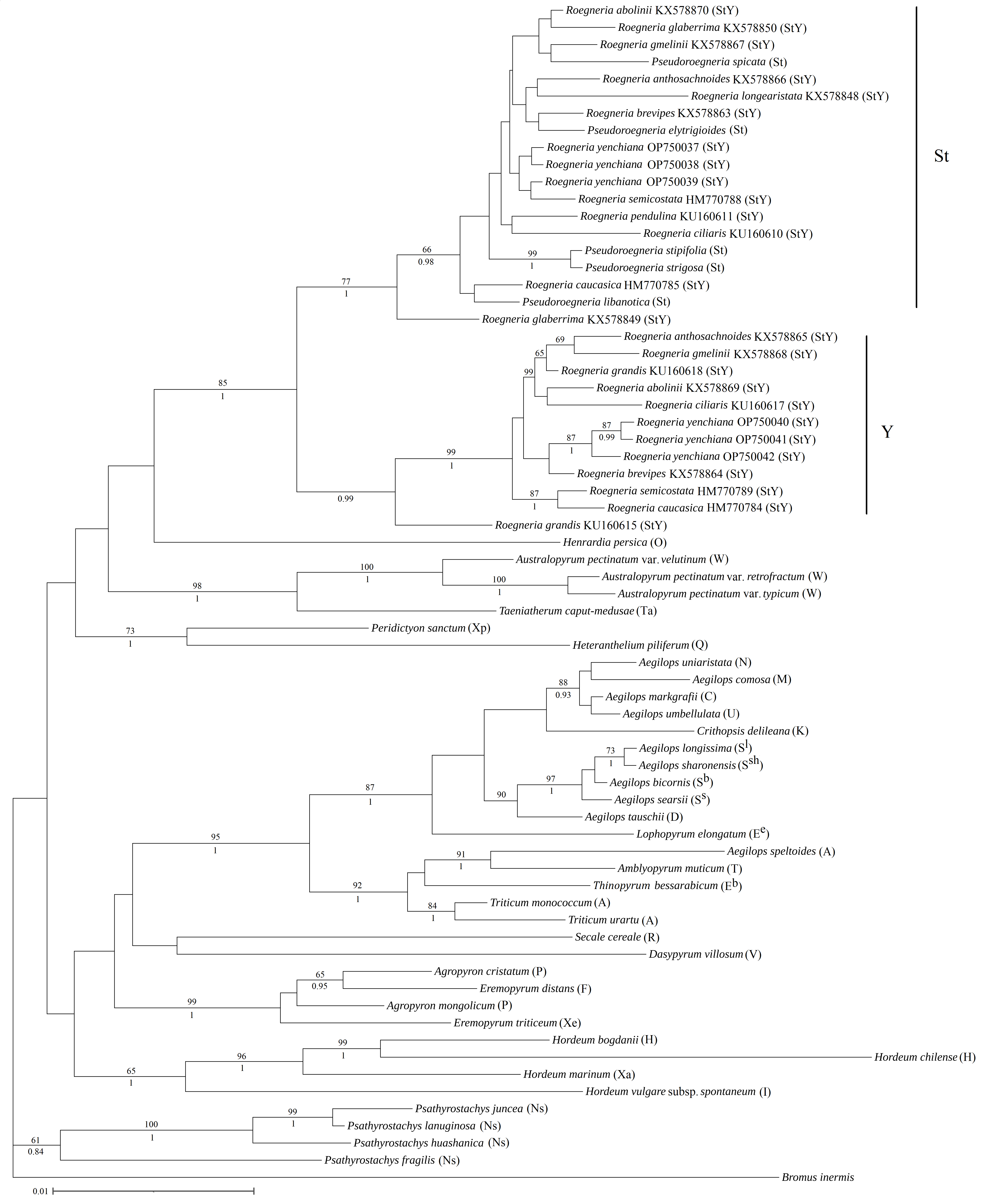

Supplement: Supplementary file 1 — Figure S1. [file ECE3-14-e11171-s005.tif]

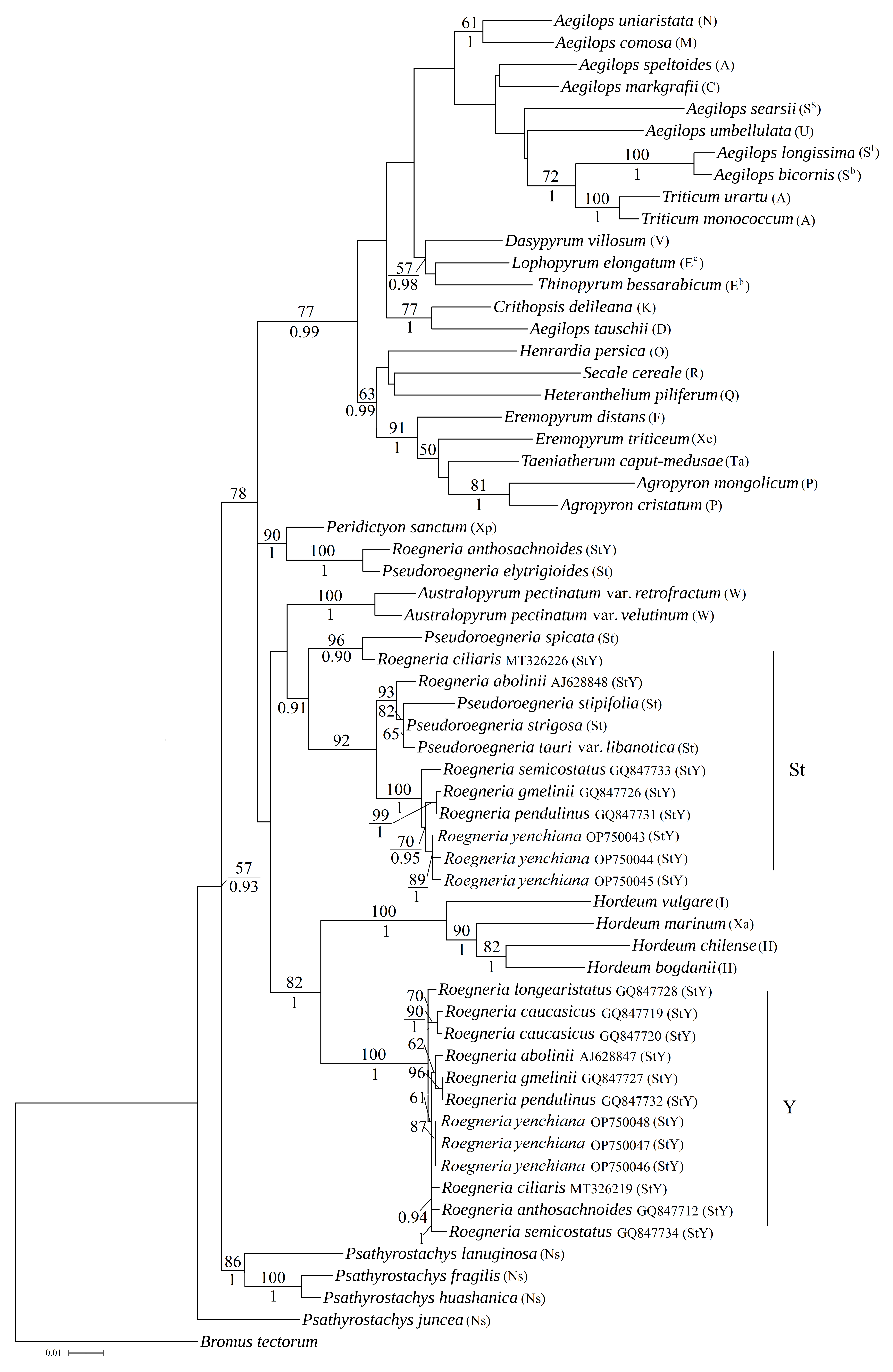

Supplement: Supplementary file 2 — Figure S2. [file ECE3-14-e11171-s002.tif]

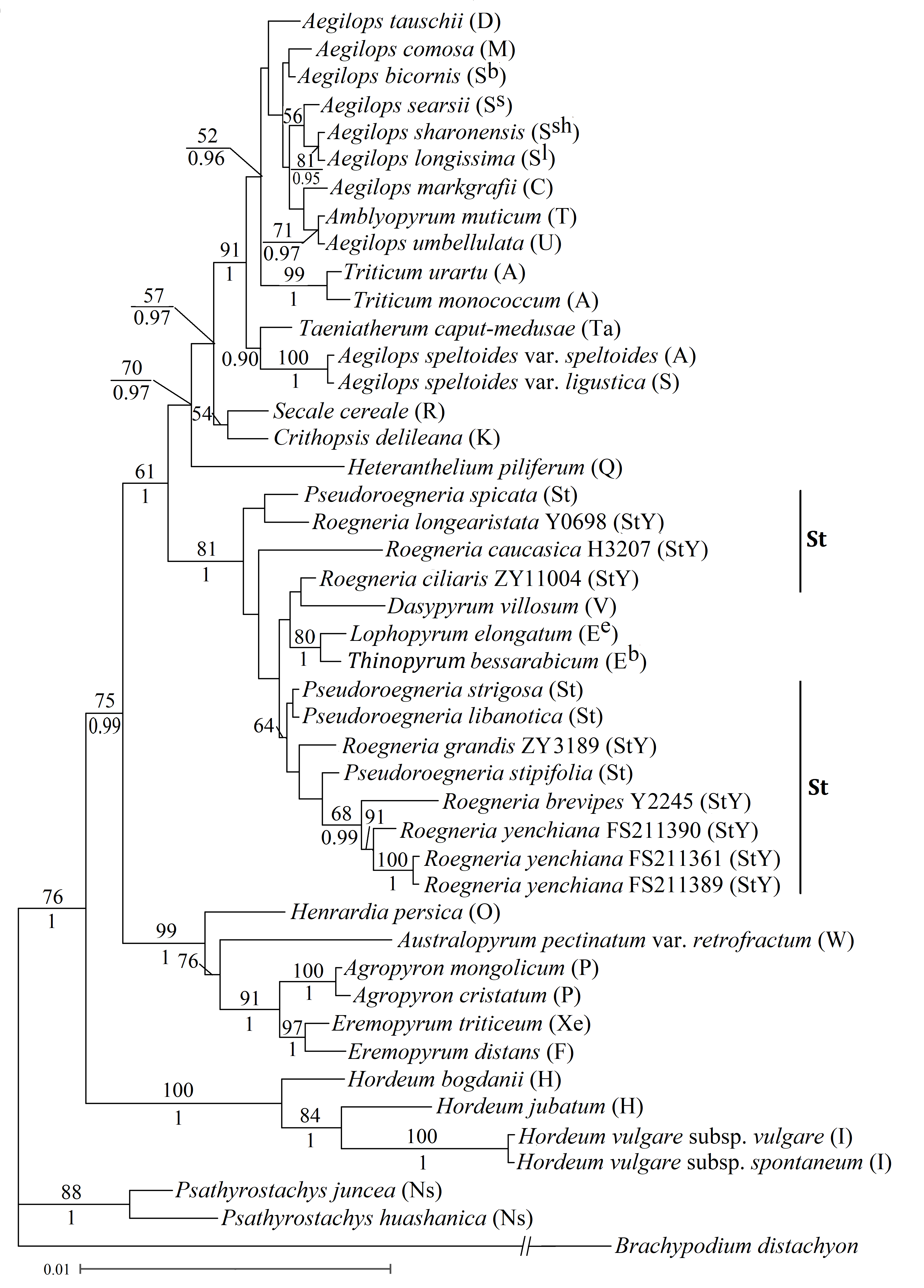

Supplement: Supplementary file 3 — Figure S3. [file ECE3-14-e11171-s006.tif]
