# Supplementary material for: Roegneria yenchiana: A new species in the Triticeae (Poaceae) from the Hengduan Mountain region
Source: Ecol Evol. 2024 Mar 17;14(3):e11171. doi: 10.1002/ece3.11171 (PMC10944672; doi:10.1002/ece3.11171)
Supplement: Supplementary file 4 — Table S1. [file ECE3-14-e11171-s004.doc]

**Table S1**. List of taxa used in this study.

| Species | Genome | | Ploidy | | GenBank Accession No. | | | | | | | | | | |
| --- | --- | --- | --- | --- | --- | --- | --- | --- | --- | --- | --- | --- | --- | --- | --- |
|  |  |  |  |  | *Acc1* | | *DMC1* | | *GBSSI* | *mat*K | | *rbc*L | | *trn*L-F | |
| ***Aegilops* L.** |  | |  | |  | |  | |  |  | |  | |  | |
| *Aegilops bicornis* | S^b^ | | 2x | | DQ290366 | | DQ247822 | | AF079265 | KJ614418 | | | KJ614418 | | KJ614418 |
| *Aegilops comosa* | M | | 2x | | -- | | DQ247824 | | AF079263 | NC046697 | | | NC046697 | | NC046697 |
| *Aegilops longissima* | S^l^ | | 2x | | DQ290369 | | DQ247830 | | AF079266 | KJ614416 | | | KJ614416 | | KJ614416 |
| *Aegilops markgrafii* | C | | 2x | | -- | | DQ247829 | | AF079262 | KY636033 | | | KY636033 | | KY636033 |
| *Aegilops searsii* | S^s^ | | 2x | | DQ290363 | | DQ247823 | | AF079264 | KJ614413 | | | KJ614413 | | KJ614413 |
| *Aegilops sharonensis* | S^sh^ | | 2x | | DQ290370 | | DQ247831 | | -- | KJ614419 | | | KJ614419 | | KJ614419 |
| *Aegilops speltoides* var*. ligustica* | S | | 2x | | -- | | -- | | -- | KJ614405 | | | KJ614405 | | KJ614405 |
| *Aegilops speltoides* var*. speltoides* | A | | 2x | | DQ290362 | | DQ247833 | | AF079267 | KJ614406 | | | KJ614406 | | KJ614406 |
| *Aegilops tauschii* | D | | 2x | | DQ290375 | | AF277235 | | AF079268 | KJ614412 | | | KJ614412 | | KJ614412 |
| *Aegilops umbellulata* | U | | 2x | | -- | | DQ247825 | | AF079269 | KY636059 | | | KY636059 | | KY636059 |
| *Aegilops uniaristata* | N | | 2x | | -- | | DQ247828 | | AF079270 | -- | | -- | | -- | |
| ***Agropyron* J. Gaertn.** |  | |  | |  | |  | |  |  | |  | |  | |
| *Agropyron cristatum* | P | | 2x | | GQ855187 | | KP211376 | | AY011002 | KY126307 | | | KY126307 | | KY126307 |
| *Agropyron mongolicum* | P | | 2x | | DQ456970 | | KP211375 | | AY011003 | MH285848 | | | MH285848 | | MH285848 |
| ***Amblyopyrum* (Jaub. & Spach) Eig** |  | |  | |  | |  | |  |  | |  | |  | |
| *Amblyopyrum muticum* | T | | 2x | | -- | | AF277243 | | -- | KY636075 | | | KY636075 | | KY636075 |
| ***Australopyrum* (Tzvelev) Á. Löve** |  | |  | |  | |  | |  |  | |  | |  | |
| *Australopyrum pectinatum* var. *typicum* | W | | 2x | | -- | | AF277252 | | -- | -- | | -- | | -- | |
| *Australopyrum pectinatum* var*. retrofractum* | W | | 2x | | DQ497807 | | AF277251 | | AF079272 | MH331642 | | | MH331642 | | MH331642 |
| *Australopyrum pectinatum* var. *velutinum* | W | | 2x | | -- | | AF277253 | | AY011004 | -- | | -- | | -- | |
| ***Crithopsis* Jaub. & Spach** |  | |  | |  | |  | |  |  | |  | |  | |
| *Crithopsis delileana* | K | | 2x | | KP940522 | | AF277240 | | GQ847707 | MH285849 | | | MH285849 | | MH285849 |
| ***Dasypyrum* (Cosson & Durieu) T. Durand** |  |  | |  | |  | |  | | | | | | | |
| *Dasypyrum villosum* | V | | 2x | | DQ456971 | | AF277238 | | AY556480 | MH285850 | | | MH285850 | | MH285850 |
| ***Eremopyrum* (Ledeb.) Jaub. & Spach** |  | |  | |  | |  | |  |  | |  | |  | |
| *Eremopyrum distans* | F | | 2x | | DQ453691 | | AF277236 | | AY011006 | MH285851 | | | MH285851 | | MH285851 |
| *Eremopyrum triticeum* | Xe | | 2x | | DQ453690 | | AF277237 | | MT112901 | MH285852 | | | MH285852 | | MH285852 |
| ***Henrardia* C. E. Hubb.** |  | |  | |  | |  | |  |  | |  | |  | |
| *Henrardia persica* | O | | 2x | | GQ228396 | | AF277255 | | MT112905 | MH285853 | | | MH285853 | | MH285853 |
| ***Heteranthelium* Hochst.** |  | |  | |  | |  | |  |  | |  | |  | |
| *Heteranthelium piliferum* | Q | | 2x | | DQ497808 | | AF277239 | | AF079277 | MH285854 | | | MH285854 | | MH285854 |
| ***Hordeum* L.** |  | |  | |  | |  | |  |  | |  | |  | |
| *Hordeum bogdanii* | H | | 2x | | KP940526 | | FJ695172 | | EU282316 | MH331641 | | | MH331641 | | MH331641 |
| *Hordeum chilense* | H | | 2x | | KP940527 | | FJ695173 | | MK045502 | -- | | -- | | -- | |
| *Hordeum jubatum* | I | | 2x | | -- | | -- | | -- | KM974741 | | | KM974741 | | KM974741 |
| *Hordeum* *marinum* | I | | 2x | | -- | | AY137397 | | AY010959 | -- | | -- | | -- | |
| *Hordeum vulgare* subsp. *spontaneum* | H | | 2x | | -- | | AF277262 | | -- | KC912689 | | | KC912689 | | KC912689 |
| *Hordeum vulgare* subsp. *vulgare* | H | | 2x | | KP940528 | | -- | | AB088761 | EF115541 | | | EF115541 | | EF115541 |
| ***Lophopyrum* (Host) Á. Löve** |  | |  | |  | |  | |  |  | |  | |  | |
| *Lophopyrum elongatum* | E^e^ | | 2x | | DQ355219 | | AF277246 | | AF079284 | MH331643 | | | MH331643 | | MH331643 |
| ***Peridictyon* O. Seberg, S. Frederiksen & C. Baden** |  | |  | |  | |  | |  |  | |  | |  | |
| *Peridictyon sanctum* | Xp | | 2x | | GQ228397 | | AF277244 | | AF079278 | -- | | -- | | -- | |
| ***Psathyrostachys* Nevski** |  | |  | |  | |  | |  |  | |  | |  | |
| *Psathyrostachys fragilis* | Ns | | 2x | | FJ449595 | | AF277261 | | AF079279 | -- | -- | | | -- | |
| *Psathyrostachys huashanica* | Ns | | 2x | | DQ335577 | | GU165826 | | KX220004 | NC_045871 | | | NC_045871 | | NC_045871 |
| *Psathyrostachys juncea* | Ns | | 2x | | DQ335578 | | EU366427 | | AF079280 | MH331640 | | | MH331640 | | MH331640 |
| *Psathyrostachys lanuginosa* | Ns | | 2x | | GQ228398 | | GU165827 | | KX220005 | -- | | -- | | -- | |
| ***Pseudoroegneria* (Nevski) Á. Löve** |  | |  | |  | |  | |  |  | |  | |  | |
| *Pseudoroegneria elytrigioides* | St | | 4x | | KC131181 | | KU160622 | | FJ602019 | -- | | -- | | -- | |
| *Pseudoroegneria libanotica* | St | | 2x | | DQ335574 | | FJ695174 | | AY360824 | KX822019 | | | KX822019 | | KX822019 |
| *Pseudoroegneria spicata* | St | | 2x | | DQ306262 | | FJ695175 | | AY010998 | MH285855 | | | MH285855 | | MH285855 |
| *Pseudoroegneria stipifolia* | St | | 2x | | DQ335576 | | FJ695176 | | EF656581 | MT385862 | | | MT385862 | | MT385862 |
| *Pseudoroegneria strigosa* | St | | 2x | | DQ335575 | | KU160624 | | EU282323 | MT385863 | | | MT385863 | | MT385863 |
| ***Roegneria* K. Koch** |  | |  | |  | |  | |  |  | |  | |  | |
| *Roegneria abolinii* | StY | | 4x | | MH046257 | | KX578870 | | AJ628848 | -- | | -- | | -- | |
| *Roegneria abolinii* | StY | | 4x | | MH046256 | | KX578869 | | AJ628847 | -- | | -- | | -- | |
| *Roegneria anthosachnoides* | StY | | 4x | | MH046265 | | KX578865 | | -- | -- | | -- | | -- | |
| *Roegneria anthosachnoides* | StY | | 4x | | MH046264 | | KX578866 | | -- | -- | | -- | | -- | |
| *Roegneria antiqua* | StY | | 4x | | MH046263 | | -- | | GQ847711 | -- | | -- | | -- | |
| *Roegneria antiqua* | StY | | 4x | | MH046262 | | -- | | GQ847712 | -- | | -- | | -- | |
| *Roegneria brevipes* | StY | | 4x | | MH046267 | | KX578864 | | -- | HQ652706 | | HQ652786 | | AY740771 | |
| *Roegneria brevipes* | StY | | 4x | | MH046266 | | KX578863 | | -- | -- | | -- | | -- | |
| *Roegneria caucasica* | StY | | 4x | | KC131179 | | HM770785 | | GQ847719 | HM770806 | | HM770839 | | AY730577 | |
| *Roegneria caucasica* | StY | | 4x | | KC131178 | | HM770784 | | GQ847720 | -- | | -- | | -- | |
| *Roegneria ciliaris* | StY | | 4x | | KP940565 | | KU160610 | | MT326219 | MK775252 | | | MK775252 | | MK775252 |
| *Roegneria ciliaris* | StY | | 4x | | KP940564 | | KU160617 | | MT326226 | -- | | -- | | -- | |
| *Roegneria glaberrima* | StY | | 4x | | MH046273 | | KX578850 | | -- | -- | | -- | | -- | |
| *Roegneria glaberrima* | StY | | 4x | | MH046272 | | KX578849 | | -- | -- | | -- | | -- | |
| *Roegneria gmelinii* | StY | | 4x | | MH046277 | | KX578867 | | GQ847726 | -- | | -- | | -- | |
| *Roegneria gmelinii* | StY | | 4x | | MH046276 | | KX578868 | | GQ847727 | -- | | -- | | -- | |
| *Roegneria grandis* | StY | | 4x | | KP940567 | | KU160615 | | -- | MN703669 | | | MN703669 | | MN703669 |
| *Roegneria grandis* | StY | | 4x | | KP940566 | | KU160618 | | -- | -- | | -- | | -- | |
| *Roegneria longearistata* | StY | | 4x | | MH046283 | | KX578848 | | GQ847728 | MN703670 | | | MN703670 | | MN703670 |
| *Roegneria longearistata* | StY | | 4x | | MH046282 | | -- | | -- | -- | | -- | | -- | |
| *Roegneria pendulina* | StY | | 4x | | KC131187 | | KU160611 | | GQ847731 | -- | | -- | | -- | |
| *Roegneria pendulina* | StY | | 4x | | KC131186 | | -- | | GQ847732 | -- | | -- | | -- | |
| *Roegneria semicostata* | StY | | 4x | | MH046291 | | HM770788 | | GQ847734 | -- | | -- | | -- | |
| *Roegneria semicostata* | StY | | 4x | | MH046290 | | HM770789 | | GQ847733 | -- | | -- | | -- | |
| *Roegneria yenchiana* | StY | | 4x | | OP747287 | | OP750040 | | OP750046 | OP747042 | | OP747063 | | OP747187 | |
| *Roegneria yenchiana* | StY | | 4x | | OP747290 | | OP750037 | | OP750043 | -- | | -- | | -- | |
| *Roegneria yenchiana* | StY | | 4x | | OP747288 | | OP750038 | | OP750047 | OP747043 | | OP747064 | | OP747188 | |
| *Roegneria yenchiana* | StY | | 4x | | OP747291 | | OP750041 | | OP750044 | -- | | -- | | -- | |
| *Roegneria yenchiana* | StY | | 4x | | OP747289 | | OP750039 | | OP750048 | OP747044 | | OP747065 | | OP747189 | |
| *Roegneria yenchiana* | StY | | 4x | | OP747292 | | OP750042 | | OP750045 | -- | | -- | | -- | |
| ***Secale*L*.*** |  | |  | |  | |  | |  |  | |  | |  | |
| *Secale cereale* | R | | 2x | | AF343516 | | KP211358 | | AY011009 | KC912691 | | | KC912691 | | KC912691 |
| ***Taeniatherum* Nevski** |  | |  | |  | |  | |  |  | |  | |  | |
| *Taeniatherum caput-medusae* | Ta | | 2x | | DQ497803 | | AF277249 | | AY011010 | MH285856 | | | MH285856 | | MH285856 |
| ***Thinopyrum* Á. Löve** |  | |  | |  | |  | |  |  | | |  | |  |
| *Thinopyrum bessarabicum* | E^b^ | | 2x | | DQ355220 | | AF277254 | | AF079283 | MH331639 | | | MH331639 | | MH331639 |
| ***Triticum* L.** |  | |  | |  | |  | |  |  | |  | |  | |
| *Triticum monococcum* | A | | 2x | | DQ290260 | | AF277250 | | AF079286 | LC005977 | | | LC005977 | | LC005977 |
| *Triticum urartu* | A | | 2x | | DQ290265 | | DQ247826 | | AF079287 | KJ614411 | | | KJ614411 | | KJ614411 |
| ***Brachypodium* Beauv.** |  | |  | |  | |  | |  |  | |  | |  | |
| *Brachypodium distachyon* | ND | | ND | | -- | | -- | | XM014897605 | EU325680 | | | EU325680 | | EU325680 |
| ***Bromus* L.** |  | |  | |  | |  | |  |  | | |  | |  |
| *Bromus inermis* | ND | | ND | | EU366392 | | MT833867 | | -- | -- | | | -- | | -- |
| *Bromus tectorum* | ND | | ND | | -- | | -- | | AY362757 | -- | | | -- | | -- |

The GenBank accession numbers without bold represent previously published sequences from the GenBank ([http://www.ncbi.nlm.nih.gov](http://www.ncbi.nlm.nih.gov/)). ND: not determined. --, unavailable sequence type.
