# Supplementary material for: Roegneria yenchiana: A new species in the Triticeae (Poaceae) from the Hengduan Mountain region
Source: Ecol Evol. 2024 Mar 17;14(3):e11171. doi: 10.1002/ece3.11171 (PMC10944672; doi:10.1002/ece3.11171)
Supplement: Supplementary file 5 — Table S2. [file ECE3-14-e11171-s001.doc]

**Table S2.** Names, sequences, and references of primers used in this study.

| Gene | primers | Sequences (5’-3’) | Reference |
| --- | --- | --- | --- |
| *Acc1* | --  -- | 5’-CCC AAT ATT TAT CAT GAG ACT TGC A-3’  5’-CAA CAT TTG AAT GAA ThC TCC ACG-3’ | Huang et al., 2002 |
| *GBSSI* | F-for  M-bac | 5’-TGC GAG CTC GAC AAC ATC ATG CG-3’  5’-GGC GAG CGG CGC GAT CCC TCG CC-3’ | Mason-Gamer, 2004 |
| *DMC* | TDMC1e10  TDMC1e15R | 5’- TGC CAA TTG CTG AGA GAT TTG-3’  5’- AGC CAC CTG TTG TAA TCT GG -3’ | Petersen and Seberg, 2000 |
| *matK* | W  9R | 5’-TAC CCT ATC CTA TCC AT-3’  5’-TAC GAG CTA AAG TTC TAG C-3’ | Hilu et al., 1999 |
| *rbcL* | --  -- | 5’-TGT CAC CAA AAA CAG AGA CT-3’  5’-TTC CAT ACT TCA CAA GCA GC-3’ | McMillan and Sun, 2004 |
| *trnL-F* | c  f | 5’-CGA AAT CGG TAG ACG CTA CG-3’  5’-ATT TGA ACT GGT GAC ACG AG-3’ | Mason-Gamer et al., 2002 |

**Reference**

Huang, S.X., Sirikhachornkit, A., Faris, J.D., Su, X.J., Gill, B.S., Haselkorn, R., Gornicki, P., 2002. Phylogenetic analysis of the acetyl-CoA carboxylase and 3-phosphoglycerate kinase loci in wheat and other grasses. Plant Mol. Biol. 48, 805-820.

Hilu, K.W., Alice, L.A., Liang, H.P., 1999. Phylogeny of Poaceae inferred from *matK* sequences. Ann, Mo. Bot. Gard. 86, 835-851.

Mason-Gamer, R.J., 2004. Reticulate evolution, introgression, and intertribal gene capture in an allohexaploid grass. Syst. Biol. 53, 25-37.

Mason-Gamer, R.J., Orme, N.L., Anderson, C.M., 2002. Phylogenetic analysis of North American *Elymus* and monogenomic Triticeae (Poaceae) using three chloroplast DNA data sets. Genome 45, 991-1002.

McMillan, E., Sun, G., 2004. Genetic relationships of tetraploid *Elymus* species and their genomic donor species inferred from polymerase chain reaction-restriction length polymorphism analysis of chloroplast gene regions. Theor. Appl. Geneti. 108, 535-542.

Petersen G, Seberg O. 2000. Phylogenetic evidence for excision of Stowaway miniature inverted-repeat transposable elements in Triticeae (Poaceae). Molecular Biology andEvolution17: 1589-1596.
