# Supplementary material for: Roegneria yenchiana: A new species in the Triticeae (Poaceae) from the Hengduan Mountain region
Source: Ecol Evol. 2024 Mar 17;14(3):e11171. doi: 10.1002/ece3.11171 (PMC10944672; doi:10.1002/ece3.11171)
Supplement: Supplementary file 6 — Table S3. [file ECE3-14-e11171-s003.doc]

**Table S3.** Features of the four matched data sets and their trees

| **Sequences** | **Total characters** | **Variable characters** | **Informative characters** | **Tree statistics** |
| --- | --- | --- | --- | --- |
|  |  |  |  | **-LogLikelihood** |
| *Acc1* | 1400 | 411 | 201 | 5890.542429 |
| *DMC1* | 959 | 343 | 158 | 4907.257991 |
| *GBSSI* | 1104 | 472 | 277 | 8424.814393 |
| combined cpDNA | 3089 | 306 | 132 | 7088.836976 |
